# All-trans retinoic acid inhibits lipopolysaccharide-induced inflammatory responses in bovine adipocytes via TGFβ1/Smad3 signaling pathway

**DOI:** 10.1186/s12917-019-1791-2

**Published:** 2019-02-01

**Authors:** Qiushi Xu, Hongdou Jia, Li Ma, Guowen Liu, Chuang Xu, Ying Li, Xinwei Li, Xiaobing Li

**Affiliations:** 1 Key Laboratory of Zoonoses Research, Ministry of Education, College of Veterinary Medicine, Jilin University, Changchun, Jilin China; 2 College of Animal Science and Veterinary Medicine, Heilongjiang Bayi Agricultural University, Daqing, China

**Keywords:** All-trans retinoic acid, TGFβ1/Smad3 signaling, Bovine adipocytes, Inflammatory responses

## Abstract

**Background:**

Dairy cows with metabolic disorder in peripartal period display high inflammatory levels. Adipose tissue is a major endocrine organ, which is closely related to systemic inflammation. Retinoic acid (RA), an active metabolite of vitamin A, has shown potential therapeutic immunomodulatory properties. The objective of the study was to examine the effect of all-trans-RA (ATRA), the biologically most active metabolite of vitamin A, on lipopolysaccharide (LPS)-induced bovine adipocytes inflammatory responses and elucidate the underlying mechanisms.

Primary cultured bovine adipocytes were treated with ATRA in the presence or absence of LPS. The treated cells were examined for the inflammatory responses and the activity of transforming growth factor beta 1 (TGFβ1) /Smad3 signaling pathway.

**Results:**

LPS treatment significantly decreased the expression levels of TGFβ1/Smad3 components and increased the content of pro-inflammatory cytokines. Treatment with ATRA could over-activate TGFβ1/Smad3 signaling pathway in bovine adipocytes and reversed the over-production of pro-inflammatory cytokines and inhibition of anti-inflammatory cytokines induced by LPS. Importantly, inhibition of TGFβ1/Smad3 signaling diminished the effects of ATRA on suppressing the proinflammatory responses induced by LPS. Furthermore, activation of TGFβ1/Smad3 signaling further extended the effects of ATRA on suppressing the proinflammatory responses on LPS stimulation.

**Conclusion:**

In conclusion, ATRA stimulates TGFβ1/Smad3 signaling pathway and further suppresses bovine adipocytes inflammatory responses induced by LPS.

**Electronic supplementary material:**

The online version of this article (10.1186/s12917-019-1791-2) contains supplementary material, which is available to authorized users.

## Background

The peripartal or transition period in dairy cattle is characterized by homeostatic changes, negative energy balance (NEB) and elevated incidence of metabolic disorders [1, 2]. Around 30 to 50% of dairy cows develop metabolic or infectious diseases during the transition period [3], which is a direct response to sudden changes in both metabolic and immune functions and may detrimental to production, health, and fertility [4]. Dairy cattle with metabolic diseases such as ketosis or fatty liver are reported to display high levels of systemic inflammation response [4], even without signs of microbial infections or otherwise determined pathological path [5]. The inflammatory state is mainly manifested as the release of pro-inflammatory cytokines. Previous studies has demonstrated that the blood concentration of cytokines, including interleukin 1 beta (IL-1β), interleukin 6 (IL-6) and tumor necrosis factor alpha (TNF-α), was significantly increased in cows with ketosis or fatty liver compared with healthy cows [4]. The secretion of these cytokines can lead to a multitude of metabolic changes, including anorexia, lipid mobilization, reduced insulin sensitivity and milk yield [6–8]. Recent experimental studies on mice have identified macrophage accumulation in adipose, which is accompanied with increased severity of metabolic disorder [7, 9]. Likewise, adipocyte-derived Th2 cytokines critically control the adipose tissue inflammation state by promoting the transcriptional program of macrophage alternative activation, which in turn providing protection against insults from inflammatory stimulants [7]. These results have clarified the importance of adipose tissue inflammation in the development of metabolic disorder. Thus, targeting adipose tissue inflammation to correct metabolic disorder are of particular importance in the treatment of peripartal diseases in dairy cattle.

Retinoic acid (RA), a naturally active vitamin A metabolite, is known for regulating a wide range of immune responses [10, 11]. Dietary vitamin A and pro-vitamin A is stored as retinyl esters or intracellularly metabolized to RA, the main active form of vitamin A [12]. There are two isoforms of RA, all-trans-RA (ATRA) and 9-cis-RA, which exert their effects on cell processes through both genomic and nongenomic mechanisms by binding to retinoid receptors and their heterodimers [13]. Adipose tissue is one of major sites of vitamin A storage and metabolism, as well as a main target of ATRA action [14, 15]. Exogenous RA treatment has also been reported to skew the immune status toward a tolerogenic status [16, 17]. Other research reports, however, have proposed a contrary role for RA in the immune response. RA has shown to execrate T cell pro-inflammatory responses [18, 19] and to worsen tissue inflammation [20]. Given this discrepancy, there is a need to gain mechanistic insights on whether RA has direct effects on adipocytes inflammatory and metabolic responses.

It is well known that TGFβ regulates both systemic immune suppression and mucosal immune regulation. The prototype TGFβ superfamily member TGFβ1 is the most abundant circulating isoform in plasma [21], and is well characterized for its role in regulating immune homeostasis [22, 23]. TGFβ1 signals via binding to type II transmembrane serine threonine kinase receptors (TGFΒR2), which in turn activate type I receptors (TGFΒR1), leading to the phosphorylation/activation of dual serine/threonine kinase receptors and transcription factors called Smads, with Smad3 serving as the principal facilitator of TGFβ signals [24, 25].

The systemic inflammation in ketosis and fatty liver cows are closely related to the inflammatory activation in adipose tissue [26]. However, whether RA alters bovine adipocytes activity of TGFβ1/Smad3 in the context of adipose tissue inflammatory responses is largely obscure. The present study provides the evidence to support that RA stimulates TGFβ1/Smad3 pathway and further suppresses bovine adipocytes inflammatory responses induced by LPS treatment.

## Results

### Culture and differentiation of bovine adipose-derived stromal vascular (SV) precursor cells

Gene expression of known biomarkers in white adipocytes were evaluated by quantitative real-time PCR (Additional file 1: Figure S1). The mRNA levels of perilipin 1 (PLIN1), a major lipid droplet coat protein and known marker of adipocytes [27], started to rise already at day 4 of differentiation in parallel with intracellular lipid accumulation. Likewise, mRNA levels of adiponectin (ADPN), a characteristic hormone of white adipocytes [28], started to rise from day 4 on. CCAAT/enhancer-binding protein-α (C/EBPα) is required both for adipogenesis and for normal adipocyte function [29]. The mRNA levels of C/EBPα was induced at day 4 and was further increased in mature adipocytes. C/EBPα promotes adipogenesis by inducing the expression of peroxisome proliferator-activated receptor gamma (PPARγ) [30]. PPAR-γ, the master regulator of adipocyte differentiation [31, 32], was induced at day 4 and was not further increased in differentiated SV cells (Additional file 1: Figure S1). Moreover, the above biomarkers in adipocyte differentiation showed no significant differences between differentiated SV cells and primary isolated mature adipocytes (Additional file 1: Figure S1). This indicated that adipose derived SV cells were fully differentiated into mature adipocytes.

### ATRA suppresses bovine adipocytes inflammatory responses

**Fig. 1:**
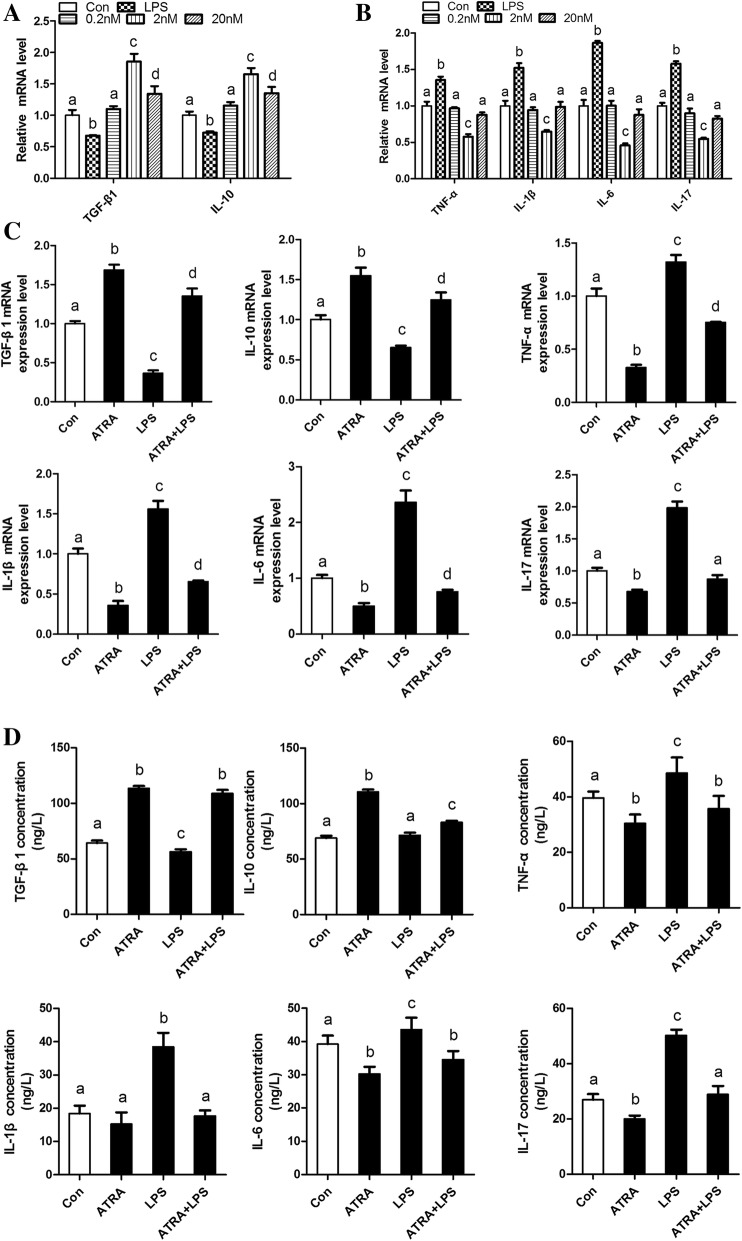
ATRA suppresses bovine adipocyte inflammatory responses. **a** qRT-PCR results showing the relative mRNA levels of anti-inflammatory cytokines (TGFβ1 and IL-10) and **(b)** pro-inflammatory cytokines (TNF-α, IL-1β, IL-6 and IL-17) in dose-response study. Adipocytes were treated with ATRA at a dose of 0.2, 2 or 20 nM (dissolved in Dimethyl sulfoxide, DMSO) 48 h. **(c)** The mRNA expression levels of TGF-β1. IL-10, TNF-α, IL-1β, IL-6, and IL-17. **d** Supernatant concentrations of TGF-β1. IL-10, TNF-α, IL-1β, IL-6, and IL-17. For all, data were presented as mean ± SEM. The same letter indicates no significant difference (*p* > 0.05), whereas different letters indicate a significant difference (*p* < 0.05)

The effect of ATRA on peripartal diseases related adipose tissue inflammation in dairy cattle remains unclear. We attempted to examine the direct effect of ATRA on bovine adipocytes inflammatory responses. Treatment with ATRA at a dose of 2 nM brought about a significant decrease in LPS-induced inflammatory responses compared with ATRA treatment at a dose of 0.2 nM and 20 nM (Fig. 1a and b). Moreover, treatment with ATRA at a dose of 2 nM appeared to cause the strongest reduction in mRNA levels of pro-inflammatory cytokines (Fig. 1b). Therefore, concentration of 2 nM ATRA was chosen as the fixed dose in our following study.

In differentiated bovine adipocytes, treatment with LPS, a powerful pro-inflammatory stimulus, caused significant increases in the mRNA levels of IL-1β, IL-6, IL-17 and TNF-α (Fig. 1c). However, treatment with ATRA in adipocytes significantly decreased the mRNA levels of IL-1β, IL-6, and TNF-α induced by LPS. Furthermore, mRNA levels of anti-inflammatory cytokines, including TGFβ1 and IL-10 was elevated significantly upon ATRA treatment (Fig. 1c). In addition, these cytokines in culture supernatant were detected using ELISA kits, treatment with ATRA also significantly inhibited these cytokines, secreted by adipocytes, induced by LPS. Upon treatment with ATRA, the effect of LPS on inducing adipocytes pro-inflammatory responses was significantly weakened (Fig. 1d).

### ATRA over-activates TGFβ1/Smad3 pathway in bovine adipocytes

**Fig. 2:**
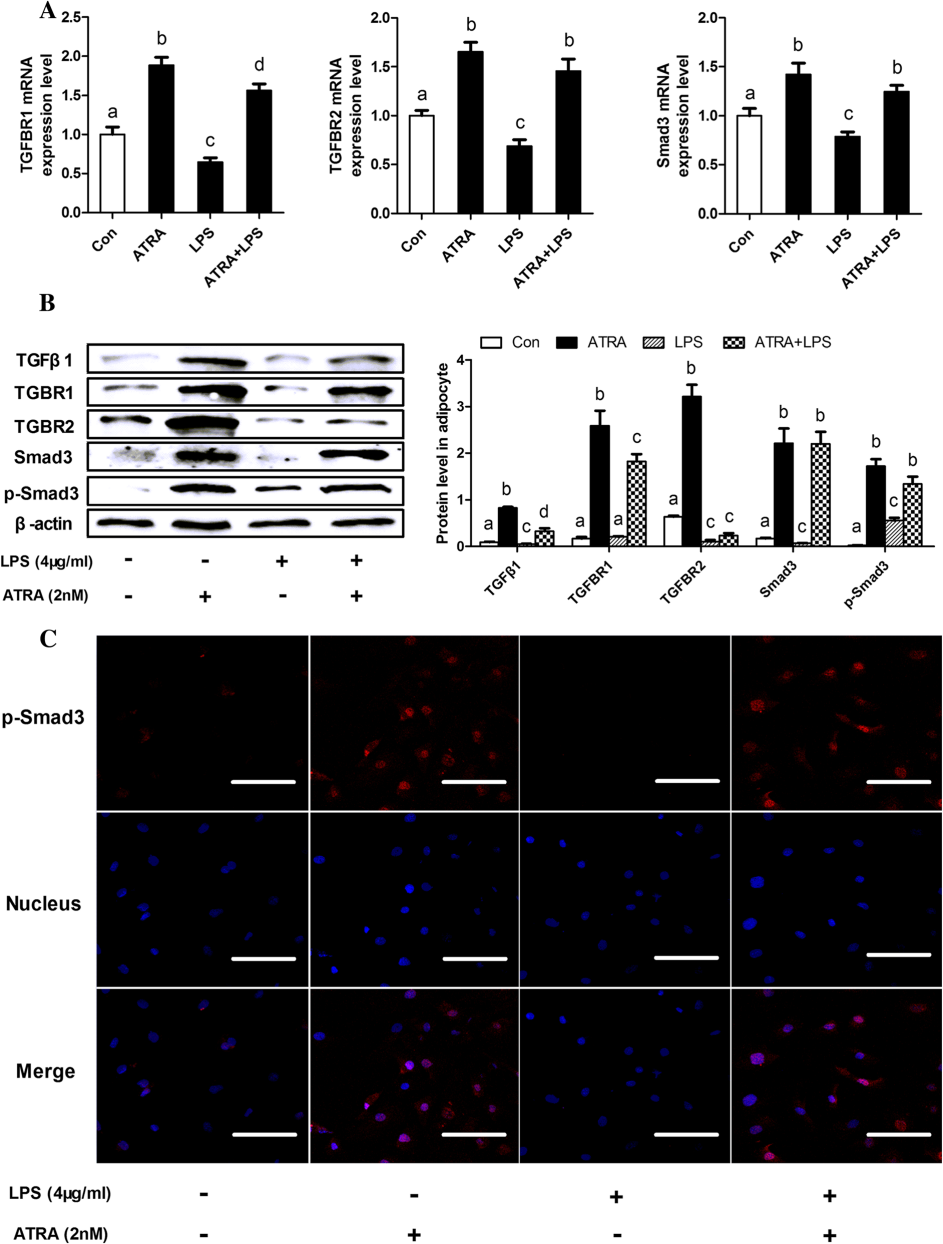
ATRA over-activates TGFβ1/Smad3 pathway in bovine adipocytes. **a** qRT-PCR results showing the relative mRNA levels of key molecules of the TGFβ1/Smad3 signaling pathway (TGFBR1, TGFBR2 and Smad3). **b** Representative western blot and its quantification showing key molecules of the TGFβ1/Smad3 signaling pathway in dairy cow adipocytes. **c** Activation of the TGF-β canonical pathway was determined by nuclear localization of p-Smad3. Bovine adipocytes were stained for p-Smad3 and nuclei were stained with DAPI. Immunofluorescence was detected using Olympus Fluoview FV1200 Laser Scanning Confocal Microscope. Scale bar = 60 μm. For all, data were presented as mean ± SEM. The same letter indicates no significant difference (*p* > 0.05), whereas different letters indicate a significant difference (*p* < 0.05)

TGFβ/Smad3 signaling has a role in glucose tolerance and energy homeostasis in mice and human [33]. Therefore, we examined the effect of ATRA on adipocytes TGFβ1/Smad3 signaling pathway. Treatment of ATRA significantly increased the mRNA and protein levels of TGFβ1/Smad3 components, including TGFβ1, TGFBR1, TGFBR2 and Smad3 when compared with the negative control group (Fig. 1c, Fig. 2a and b). However, the mRNA and protein levels of TGFβ1/Smad3 components were decreased by LPS stimulation. Importantly, treatment of ATRA reversed LPS-induced decrease on the mRNA and protein levels of TGFβ1/Smad3 components and phosphorylation of Smad3 (Fig. 1c, Fig. 2a and b).

To evaluate the transcriptional activity of p-Smad3 in bovine adipocytes after ATRA treatment, immunocytofluorescence was performed. The results showed that ATRA increased p-Smad3 translocation to the nucleus when compared with the control group (Fig. 2c). However, it was significantly decreased in LPS treatment (Fig. 2c). Of importance, treatment of ATRA reversed the effect of LPS on decreasing p-Smad3 translocation to the nucleus (Fig. 2c). These results indicate that ATRA can increase p-Smad3 transcriptional activity in bovine adipocytes. Taken together, these results suggest that ATRA has a direct effect on stimulating adipocytes activity of TGFβ1/Smad3 signaling.

### TGFβ1/Smad3 suppresses bovine adipocytes inflammatory responses

**Fig. 3:**
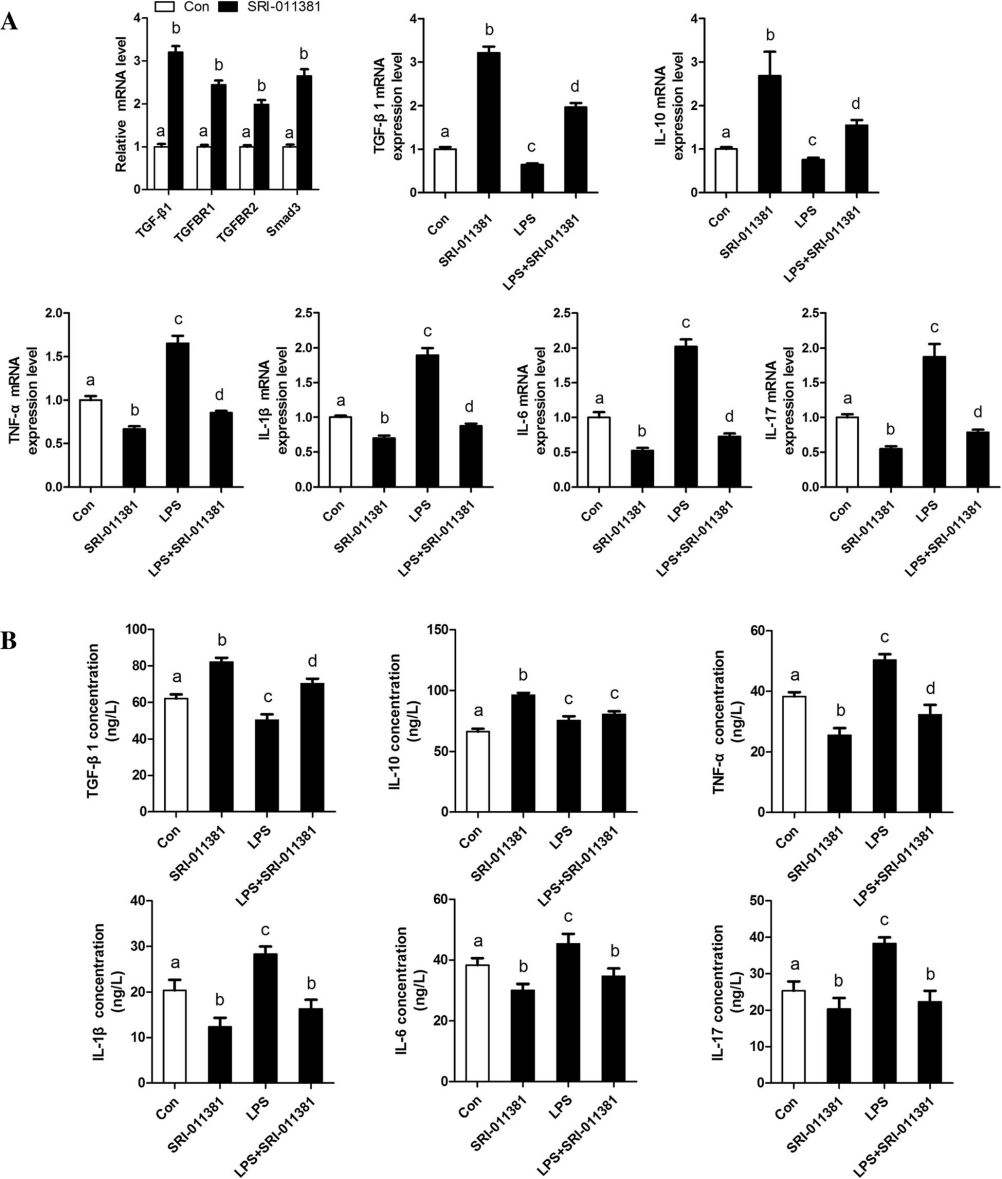
Activation of TGFβ1/Smad3 signaling pathway by SRI-011381 suppresses bovine adipocytes pro-inflammatory responses. **a** qRT-PCR results showing the relative mRNA levels of key molecules of the TGFβ1/Smad3 signaling pathway (TGF-β1, TGFBR1, TGFBR2 and Smad3). and inflammatory cytokines (TGF-β1. IL-10,TNF-α, IL-1β, IL-6 and IL-17). **b** Supernatant concentrations of TGF-β1. IL-10, TNF-α, IL-1β, IL-6, and IL-17. For all, data were presented as mean ± SEM. The same letter indicates no significant difference (*p* > 0.05), whereas different letters indicate a significant difference (*p* < 0.05)

**Fig. 4:**
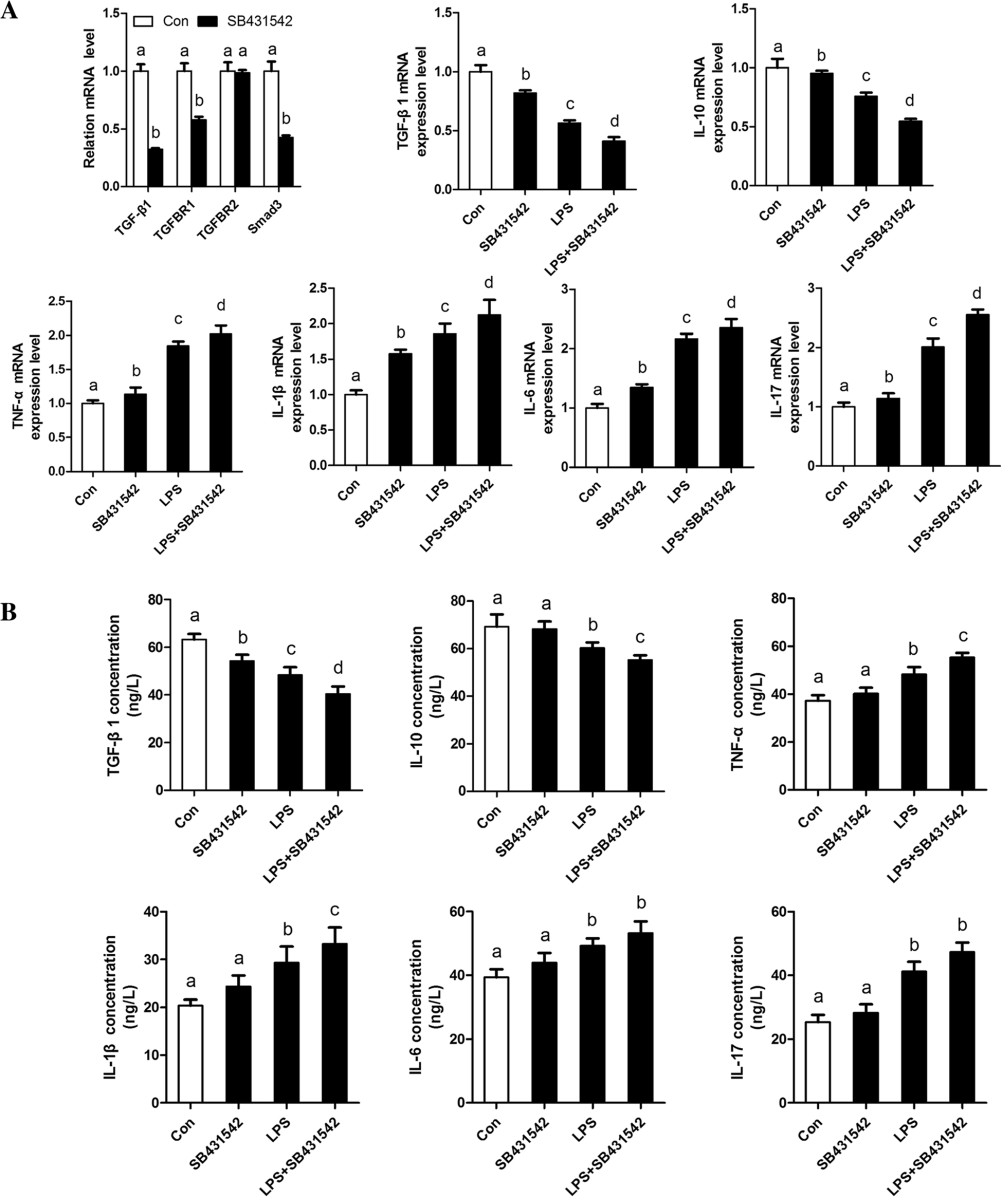
Inhibition of TGFβ1/Smad3 signaling pathway by SB431542 promotes bovine adipocytes pro-inflammatory responses. **a** qRT-PCR results showing the relative mRNA levels of key molecules of the TGFβ1/Smad3 signaling pathway (TGF-β1, TGFBR1, TGFBR2 and Smad3). and inflammatory cytokines (TGF-β1. IL-10,TNF-α, IL-1β, IL-6 and IL-17). **b** Supernatant concentrations of TGF-β1. IL-10, TNF-α, IL-1β, IL-6, and IL-17. For all, data were presented as mean ± SEM. The same letter indicates no significant difference (*p* > 0.05), whereas different letters indicate a significant difference (*p* < 0.05)

To better understand the effect of TGFβ1/Smad3 signaling on adipose tissue inflammation, bovine adipocytes were treated with TGFβ1 signaling inhibitor (SB431542) and agonist (SRI-011381), respectively. Our data further indicate that SRI-011381 significantly inhibited LPS-induced expression and secretion of pro-inflammatory cytokines, meanwhile, accelerated the expression and secretion of anti-inflammatory cytokines in bovine adipocytes (Fig. 3). However, bovine adipocytes following treatment with SB431542 further exacerbated LPS-induced inflammatory responses in bovine adipocytes (Fig. 4). Thus, our data indicate that TGFβ1/Smad3 signaling has a direct effect on suppressing bovine adipocytes inflammatory responses.

### ATRA suppresses bovine adipocytes inflammatory response dependent on TGFβ1/Smad3 signaling activity

**Fig. 5:**
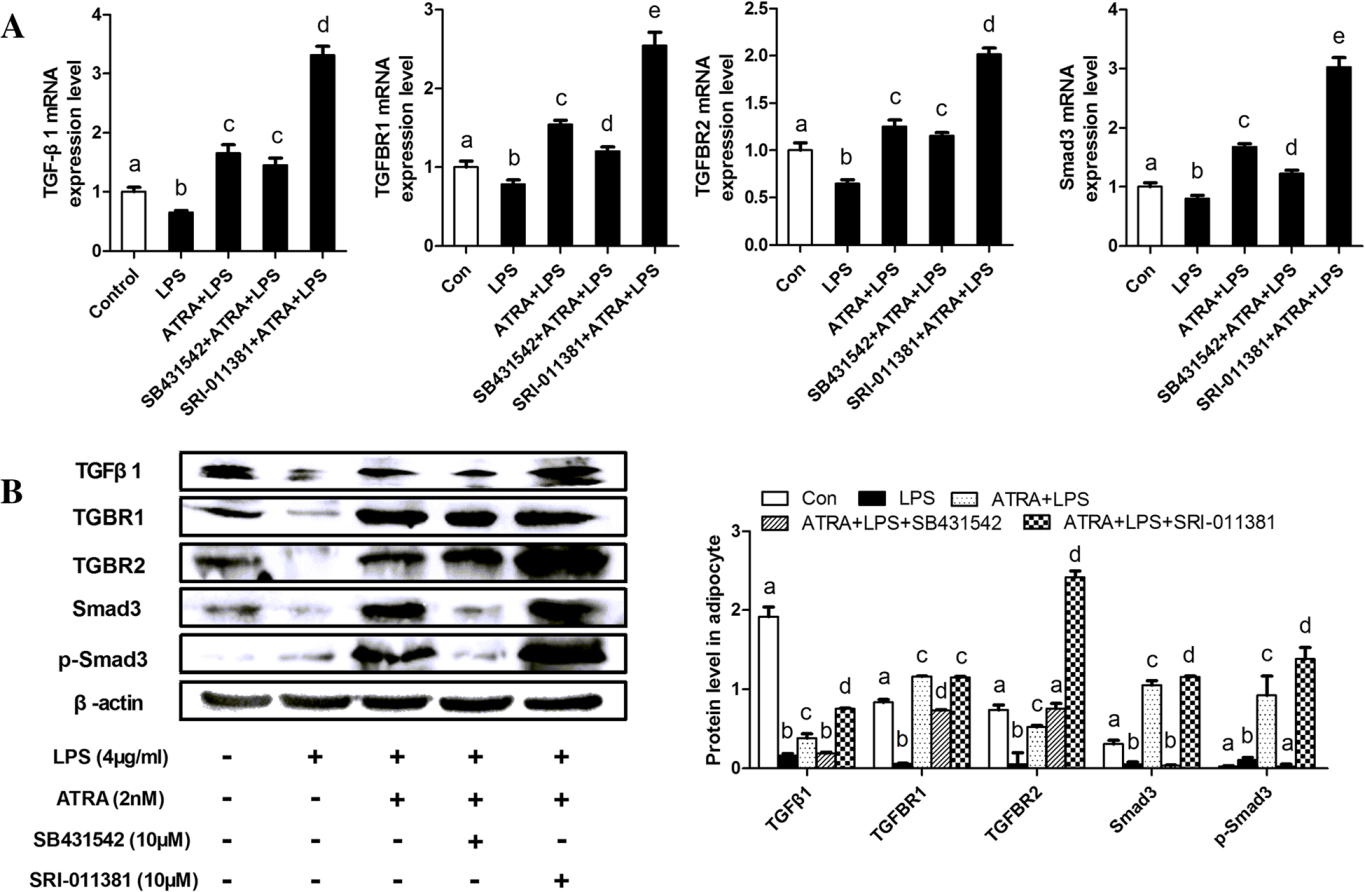
ATRA suppresses bovine adipocytes inflammatory response dependent on TGFβ1/Smad3 activity. **a** qRT-PCR results showing the relative mRNA levels of key molecules of the TGFβ1/Smad3 signaling pathway (TGFβ1, TGFBR1, TGFBR2 and Smad3). **b** Representative western blot and its quantification showing key molecules of the TGFβ1/Smad3 signaling pathway in dairy cow adipocytes. For all, data were presented as mean ± SEM. The same letter indicates no significant difference (*p* > 0.05), whereas different letters indicate a significant difference (*p* < 0.05)

We demonstrated that ATRA activated TGFβ1/Smad3 signaling pathway and suppressed bovine adipocytes inflammatory response. To further confirm this finding, we examined the effect of ATRA on LPS-induced inflammatory responses at the presence of TGFβ1/Smad3 signaling inhibitor (SB431542) and agonist (SRI-011381) in bovine adipocytes. Treatment with ATRA increased TGFβ1/Smad3 signaling activity in bovine adipocytes (Fig. 5). However, treatment with SB431542 could inhibit the stimulatory effect of ATRA on TGFβ1/Smad3 components (Fig. 5). Furthermore, the effect of ATRA was further enhanced in SRI-011381-treated bovine adipocytes (Fig. 5). Our data further indicate that inhibition of TGFβ1/Smad3 signaling pathway significantly impaired the ability of ATRA to suppress LPS-induced bovine adipocytes inflammation.

**Fig. 6:**
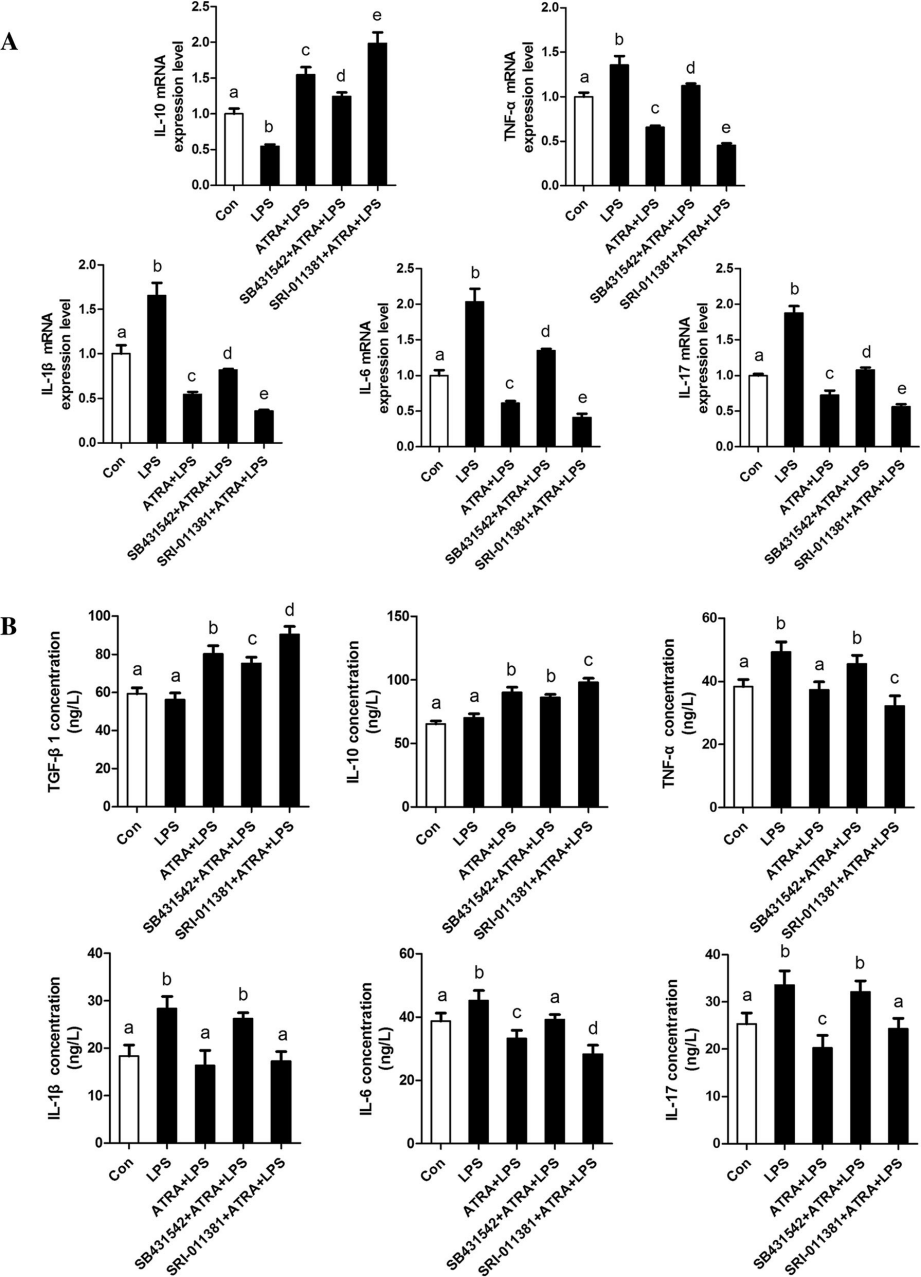
Inhibition of TGFβ1/Smad3 signaling pathway impair the effect of ATRA on suppressing proinflammatory responses. **a** qRT-PCR results showing the relative mRNA levels of key molecules of the TGFβ1/Smad3 signaling pathway (TGF-β1, TGFBR1, TGFBR2 and Smad3). and inflammatory cytokines (TGF-β1. IL-10,TNF-α, IL-1β, IL-6 and IL-17). **b** Supernatant concentrations of TGF-β1. IL-10, TNF-α, IL-1β, IL-6, and IL-17. For all, data were presented as mean ± SEM. The same letter indicates no significant difference (*p* > 0.05), whereas different letters indicate a significant difference (*p* < 0.05)

Next, we examined the anti-inflammatory effect of ATRA. The mRNA levels of pro-inflammatory cytokines (IL-1β, IL-6, IL-17 and TNF-α) were significantly reduced by treatment with ATRA under basal conditions (in the absence of LPS) compared with control (in the absence of ATRA), meanwhile, anti-inflammatory cytokines including TGFβ1 and IL-10 were significantly increased by treatment with ATRA under basal conditions compared with control (Fig. 5a, Fig. 6). In contrast, the mRNA levels and synthesis of pro-inflammatory cytokines in SB431542-treated cells were increased or remained high upon treatment with ATRA under LPS-stimulated condition (Fig. 6). Furthermore, treatment with SRI-011381 further enhanced the anti-inflammatory effect of ATRA on inhibiting the production of pro-inflammatory cytokines and simultaneously promoted the production of anti-inflammatory cytokines (Fig .5a, Fig. 6). Taken together, these results suggest that intact TGFβ1/Smad3 signaling is required for ATRA to fully suppress LPS-induced inflammatory responses in bovine adipocytes.

## Discussion

Much of the research devoted to understanding adipose tissue development is currently performed in vitro. Several cell culture models, including preadipocyte cell lines and primary culture of adipose-derived SV precursor cells, are commonly used to study molecular and cellular events and regulatory influences on adipocyte proliferation and differentiation [34]. Besides, the specific method of isolating mature adipocytes from adipose tissue is also established [35]. However, due to the small amount of cells, it is impossible to study the long-term effects of various biological and physicochemical factors through this cell model [35, 36]. Primary cultures of adipose derived SV cells more closely reflect the in vivo characteristics of the tissue from which they are derived [34]. As a major primary cell model for studying adipose tissue development and metabolism in vitro, adipose derived SV cells were chosen as the cell model in our research.

The impact of RA on glucose tolerance, insulin sensitivity and oxidative phosphorylation in lean and obese animals has been well established [37–41]. However, it remains unknown whether RA suppresses bovine adipose tissue inflammation and may thereby contribute to the treatment of peripartal metabolic diseases in dairy cattle. Because the discrepancy of ATRA actions on adipose tissue inflammation appears to be associated with various factors, including the cell strains, the doses and the paths of ATRA delivery, the present study sought to determine the effect of ATRA on adipocytes. The present study validated a direct effect of ATRA on suppressing bovine adipocytes proinflammatory responses. Furthermore, the present study revealed an inhibitory effect of ATRA on the synthesis and release of proinflammatory cytokines via TGFβ1/Smad3 signaling pathway. Therefore, the present study provided the evidence to support ATRA stimulation of adipocyte TGFβ1/Smad3 as a novel mechanism by which ATRA suppress bovine adipose tissue inflammation.

The adipose tissue was once thought to be an inert tissue used solely for storage of excess energy. Identification of many bioactive proteins secreted by adipose tissue assigned the adipocyte a central role in the pathophysiology of insulin resistance and the metabolic syndrome [42]. During the transition period, cows have a natural genetic drive to mobilize adipose tissue [43]. Although lipolysis ensures an adequate supply of energy around parturition, when intense and protracted, it predisposes cows to high levels of systemic inflammation response by limiting adipose tissue capacity for energy buffering and contributing to chronically increased pro-inflammatory cytokines secretion [44, 45]. During the development of metabolic disorder, adipose tissue is considered to be a major source of inflammatory mediators, such as TNF-α, IL-1β, IL-6 and IL-17, which negatively affect adipocyte function [46]. In this study, our data indicated that treatment of ATRA reversed the negative effect of LPS-induced secretion of pro-inflammatory cytokines. Similar immune suppressive and anti-inflammatory properties of RA have been shown in the therapeutic for inflammatory bowel diseases [47, 48] and neuroinflammatory disorders [49]. Thus, the above results led us to further investigate the mechanism of ATRA on suppressing bovine adipocytes inflammation responses.

Obese individuals have shown effective immunoregulation to counter chronic obesity-related inflammation through the increased production of the anti-inflammatory cytokines TGFβ and IL-10 in adipose tissue [50]. Similarly, our results showed that ATRA significantly increased the production TGFβ1 and IL-10 to suppress LPS-induced inflammatory responses in bovine adipocytes. Notably, it is a novel finding that treatment with ATRA increased bovine adipocytes activity of TGFβ1/Smad3 signaling. As shown in Naive CD4+ T cells, RA shows the ability of increases Foxp3+ Regulatory T cells and inhibits development of pro-inflammatory T cell by enhancing TGFβ-dependent Smad3 signaling [51]. In the present study, ATRA increased adipocyte TGFβ1/Smad3 signaling activity in the absence of TGFβ1 administration. Considering this, ATRA is likely capable of stimulating TGFβ1/Smad3 signaling in both TGFβ-dependent and TGFβ-independent manners. Regardless, increased expression of TGFβ1/Smad3 components was correlated with the effect of ATRA on suppressing bovine adipocytes proinflammatory responses, suggesting the participation of TGFβ1/Smad3 signaling in the anti-inflammatory effect of ATRA. In adipose tissue, the TGFβ/Smad3 signaling has been demonstrated as a metabolic regulator that links the development of insulin resistance, lipid metabolism and inflammatory responses [25, 52]. In the current experiment, activation of TGFβ1/Smad3 signaling suppressed LPS-induced inflammatory responses and inhibited the production of pro-inflammatory cytokines, whereas inhibition of TGFβ/Smad3 signaling further exacerbated LPS-induced inflammatory responses. Moreover, in Mouse Embryonic Fibroblasts (MEFs), researchers use chromatin immunoprecipitation to map 354 RA receptor (RAR) binding loci in MEFs, most of which were similarly occupied by the RARα and RARγ receptors. Only a subset of the genes associated with these loci are regulated by RA, among which are several critical components of the TGF-β pathway [53]. Because of these results, it is conceivable that ATRA stimulation of TGFβ1/Smad3 signaling may serve as a mechanism by which ATRA suppresses bovine adipocytes pro-inflammatory responses.

The participation of TGFβ1/Smad3 in the anti-inflammatory effect of ATRA was further supported by the results from adipocytes upon treatment of TGFβ1/Smad3 signaling inhibitor (SB431542) and agonist (SRI-011381). In the present study, increased pro-inflammatory responses induced by LPS were confirmed in SB431542-treated adipocytes compared with those in control adipocytes. Importantly, ATRA did not effectively suppress the LPS-induced proinflammatory responses in SB431542-treated adipocytes as did it in control adipocytes. Furthermore, treatment of SRI-011381 further expanded the effect of ATRA on suppressing LPS-induced pro-inflammatory responses. As previously demonstrated by Luo and coworkers, TGFβ1 was capable of enhancing the regenerative capacity of a xenogeneic acellular nerve matrix (XANM)- adipose-derived mesenchymal stem cells (ADSCs) graft, which was due to, in large part, decreased inflammation [33]. In contrast, TGFβ1 knockout mice showed severe autoimmune disorders, leading to decreased life spans and generally die from multifocal inflammation [54]. Based on this, it is very likely that intact TGFβ1/Smad3 is required for a metabolic environment in which ATRA is able to effectively suppress the proinflammatory responses.

## Conclusions

In summary, ATRA appears to act through simulating TGFβ1/Smad3 signaling pathway to suppress LPS-induced bovine adipocytes pro-inflammatory responses. The potential therapeutic role of ATRA on bovine adipose tissue inflammation can contribute to the treatment of peripartal metabolic diseases in dairy cattle.

## Methods

### Isolation of cattle adipose-derived SV precursor cells

The cattle adipose derived SV cells were isolated as described in the study of Zhang et al. [26]. Healthy day-old Holstein calves were treated with anesthesia and adipose tissue from the peritoneal omentum and mesentery was obtained by surgery under sterile conditions. Cattle received flunixin meglumine (Schering-Plough Sante Animale; 1.1 mg/kg IV every 12 h) postoperatively for 5 days. Monitoring consisted of twice daily examination including incisional inspection, behavior, appetite, urination, and defecation. Cattle were confined until suture removal 10–14 days following surgery and then returned to the farm and routine management in Ground dairy farm (Ground Dairy Industry Co., Ltd., Changchun, Jilin, China). All sections of this report adhere to the ARRIVE Guidelines for reporting animal research [55]. A completed ARRIVE guidelines checklist is included in Additional file 2: Checklist S1.The resulting tissue was rinsed in sterile phosphate-buffered saline (PBS) containing penicillin (2500 U/ml) and streptomycin (2500 mg/ml) to remove adherent blood. The fascia and blood vessels visible in the tissue were carefully peeled away, the resulting tissue was cut into small pieces of approximately 1 mm^3^, and collagenase type I (Sigma-Aldrich, St. Louis, MO, USA) digestion solution (final concentration 1 mg/mL) was added at 37 °C and allowed to incubate for 1.5 h. The mixture was removed from the untreated tissue through a 40 μm cell filter and the filtrate which contained the collagenase digest, and stromal-vascular (SV) cells were separated from adipocytes and medium by centrifugation at 1000 rpm for 10 min. The adipocytes were then incubated in a volume of 5 mL for RNA isolation. The SV cells are defined as those cells isolated by collagenase digestion that do not float. The residual erythrocytes were removed by adding ACK lysis buffer (Beyotime Institute of Biotechnology, Jiangsu, China) into the precipitate and centrifuging at 1000 rpm for 10 min. The supernatant was discarded, and the pellet was resuspended with basic culture medium (BCM), which was DMEM/F12 (HyClone, Logan, Utah, USA) with 10% fetal bovine serum (HyClone, Logan, Utah, USA) and 1% penicillin/streptomycin (HyClone, Logan, Utah, USA). After cell counting, the cell suspension was adjusted to a concentration of 1 × 10^4^/cm^2^ and inoculated in T25 flasks (Corning Costar Corp., Cambridge, MA, USA). The culture was then incubated at 37 °C and 5% CO_2_ in a cell incubator for 24 h, and the medium was replaced to remove nonadherent cells and tissue residues. Finally, the BCM was replaced every other day.

### Experimental cell culture and treatment

Initially, cells were resuspended in BCM and 1.8 × 10^5^ cells were seeded per six wells (Corning Costar Corp., Cambridge, MA, USA). After 5 days, when pre-confluence was reached to approximately 70%, the BCM was discarded and the BCM was replaced by differentiation culture medium 1 (DCM1), containing 10% fetal bovine serum (FBS) (HyClone, Logan, Utah, USA), 1% penicillin–streptomycin, 0.5 mM 3-Isobutyl-1-methylxanthin (IBMX) (I-7018 Sigma-Aldrich, St. Louis, MO, USA), 1 μM dexamethasone (DEX) (D-4902 Sigma-Aldrich, St. Louis, MO, USA) and 1 μg/ml insulin (I-5500 Sigma-Aldrich, St. Louis, MO, USA) in 500 ml BCM. The primary cultured SV cells were cultivated with DCM1. After 96 h, DCM1 was replaced by growth medium containing 10% FBS, 1% penicillin–streptomycin and 1 μg/ml insulin in 500 ml DMEM. The medium was renewed every 2 days until visible lipid drops appeared in the cell, indicating that the cells have completed differentiation. This period lasted approximately 10 days. After differentiation, the amount of mature adipocytes was 4.0 × 10^5^ per six wells plate. ATRA treated and -untreated adipocytes were harvested after 48 h. Cells were cultured for 48 h either in the presence or absence of LPS of 4 μg/ml lipopolysaccharide (LPS) from *Escherichia coli* O55:B5 (Sigma catalog no. L 6529).

In the dose-response study, the differentiated adipocytes were treated with ATRA at a dose of 0.2, 2 or 20 nM (dissolved in Dimethyl Sulfoxide, DMSO) or DMSO for 48 h to harvest total RNA samples and cell supernatant. To analyze the activity of TGFβ1/Smad3 signaling, adipocyte RNA was subjected to reverse transcription and real-time PCR to quantify TGFβ1, TGFBR1, TGFBR2, Smad3 and p-Smad3 mRNA levels, whereas adipocyte lysates were subjected to Western blot analysis to examine TGFβ1, TGFBR1, TGFBR2, Smad3 and p-Smad3 amount. In addition, adipocyte RNA and the culture supernatant were used to examine the levels of inflammatory cytokines, including IL-1β, IL-6, IL-10, IL-17 and TNF-α. Details were described in the pertinent assays.

To examine the involvement of TGFβ1/Smad3 signaling in ATRA actions, TGFβ1/Smad3 signaling inhibitor (SB431542) and agonist (SRI-011381) were used with ATRA to examine the changes in the inflammatory responses and TGFβ1/Smad3 signaling activity. After differentiation, the cells were pretreated with SB431542 (10 μM) or SRI-011381 (10 μM) for 3 h. After that, adipocytes were treated with ATRA (2 nM) or DMSO for 48 h in the absence or presence of LPS (4 μg/mL) for the last 6 h to harvest total RNA samples and protein lysates. Protein lysates were harvested and used to examine TGFβ1/Smad3 signaling using Western blot analysis.

### Oil red O staining

After induction of differentiation described above, cells were washed three times in phosphate buffered saline (PBS), fixed in 10% formalin for 15 min and washed a further three times in PBS. Subsequently, cells were washed in 60% (*v*/v) isopropanol for 2 min, stained with 0.5% (*w*/*v*) oil red O solution for 15 min, washed with 60% isopropanol and subsequently with PBS, and then counter-stained with hematoxylin prior to microscopy. After induction of differentiation, the representative images are shown in Additional file 1: Figure S1.

### Western blot analysis

Adipocyte lysates were prepared in a lysis buffer containing 50 mM HEPES (pH 7.4), 10 mM EDTA, 50 mM sodium pyrophosphate, 0.1 M sodium fluoride, 10 mM sodium orthovanadate, 2 mM phenylmethylsulfonyl fluoride, 10 μg/ml aprotinin, 10 μg/ml leupeptin, 2 mM benzamidine and 1% Triton X-100. After protein electrophoresis and transfer, immunoblots were performed using rabbit anti-serum as primary antibody at a 1:1000 dilution. This dilution was used for each of the primary antibodies used for the present study. After washing, the blot was incubated with a 1:10,000 dilution of goat anti-rabbit horseradish peroxidase–conjugated secondary antibody followed by a chemiluminescent kit (Immobilon Western; EMD Millipore, Billerica, MA, USA) as previously described [56]. β-actin was used as a loading control. The maximum intensity of each band was quantified using ImageJ software. Ratios of TGF-β1, TGFBR1, TGFBR2, Smad3 and p-Smad3 were normalized to β-actin. Antibodies against TGF-β1 (ab9758). TGFBR1 (ab31013), TGFBR2 (ab186838), SMAD3 (ab227223) and p-Smad3 (ab118825) were products of Abcam (Cambridge, UK). Anti-rabbit IgG antisera were products of Cell Signaling Technology, Inc. (Danvers, MA, USA).

### RNA isolation, reverse transcription and quantitative real-time PCR

The total RNA was isolated from the adipocytes described above using TRIPURE reagent (Roche) according to the manufacturer’s instructions and reverse transcription was performed using the GoScript Reverse Transcription System (Promega), and real-time PCR analysis was performed using SYBR Green (LightCycler 480 system; Roche). Reverse transcription was performed at 42 °C for 60 min with 2 μg total RNA in 25 μl volume. The conditions used for real-time PCR were as follows: 95 °C for 3 min, followed by 40 cycles of 95 °C for 15 s and 60 °C for 1 min. All reactions were run in triplicate. The mRNA levels were analyzed for ADPN, C/EBPα, IL-1β, IL-6, IL-10, IL-17, PLIN1, PPARγ, TNF-α, TGFBR1, TGFBR2, Smad3 and TGFβ-1. The fold change for each target gene relative to the control group was calculated using the 2^-ΔΔCt^ method [57]. The information of the primer sequences are summarized in Table 1.

**Table 1:** Primers information for qRT-PCR in the study

Gene	Primer sequences(5′-3′)	Size(bp)
ADPN	F GGAATGACAGGAGCTGAAGG R CGAATGGGTACATTGGGAAC	143
C/EBPα	F TGGACAAGAACAGCAACGAG R TCACTGGTCAACTCCAGCAC	127
IL-6	F GCAAGGAGACACTGGCAGAA R CTCCAGAAGACCAGCAGTGG	122
IL-10	F TGAAGGACCAACTGCACAGC R TGTGGCATCACCTCTTCCAG	121
IL-17	F TGAGTCTGGTGGCTCTTGTG R GGAGAGTCCAAGGTGAGGTG	180
PLIN1	F CACCTGTGAGTGCTTCCAGA R AGCTACGAACTGGGTGGACA	163
PPARγ	F CGAGAAGGAGAAGCTGTTGG R TCAGCGGGAAGGACTTTATG	122
TNF-α	F TCTTCTGCCTGCTGCACTTC R CGGCTTGTTACTTGAGGCTTG	128
TGFBR1	F ATGGTTCCGTGAAGCAGAGA R CTGACACCAACCAGAGCTGA	119
TGFBR2	F AAGCAGAACACGTCTGAGCA R CAGGATGTTCTCGTGCTTGA	126
Smad3	F AGCTGACACGGAGGCATATC R GCCATAGCGCTGGTTACAGT	125
TGF-β1	F GAACTGCTGTGTTCGTCAGC R TCCAGGCTCCAGATGTAAGG	126
GAPDH	F AAGTTCAACGGCACAGTCAA R TACTCAGCACCAGCATCACC	121

### Immunocytofluorescence

Primary cultured SV cells were seeded on glass coverslips in a 6-well cell culture plate and induced to differentiate when they were approximately 70% confluent. After differentiation, LPS, ATRA treatment was performed as described above. Successively, the glass coverslips were washed with 0.01 M PBS, fixed with 4% *w*/*v* paraformaldehyde for 20 min at room temperature, subjected to antigen recovery with EDTA-Na_2_ (95 °C, 5 min) and punched with 0.1% Triton X-100 (Sigma-Aldrich, St. Louis, MO, USA) for 10 min. After washing again, the cells were incubated overnight at 4 °C with primary antibody p-Smad3 (ab118825; Abcam, Cambridge, UK) diluted 1:200 with goat serum, then treated with goat anti-rabbit IgG conjugated with Cy3 (Beyotime Institute of Biotechnology, Jiangsu, China) at 1:200 in PBS for 30 min at room temperature and re-stained with Hoechst 33258 (Beyotime Institute of Biotechnology, Jiangsu, China). The coverslips were observed and photographed using a laser scanning confocal microscope (Fluoview FV1200, Olympus, Japan).

### Enzyme-linked immunosorbent assay (ELISA)

After differentiation and treatment of cells in 6-well cell culture plates, the supernatant of the culture medium was collected by centrifugation. The levels of IL-1β, IL-6, IL-10, IL-17, TNF-α and TGFβ-1 in the supernatant were measured by an ELISA kit (IL-1β: orb437230; IL-10: orb437130; TGFβ-1: orb403324; Biorbyt Ltd. Waterbeach, Cambridge, UK and IL-6: ml064296; IL-17: ml67108; TNF-α: ml024586; Shanghai Enzyme-linked Biotechnology Co., Ltd., Shanghai, China) according to the manufacturer’s instructions.

### Statistical analysis

All experiments were conducted in 3 separate cell preparations from 3 calves using at least 3 replicates per treatment. Numeric data are presented as means ± SEM (standard error of the mean). Two-tailed ANOVA and/or Student’s t-tests were used for statistical analyses. All data were analyzed by Statistical Package for the Social Sciences (SPSS) 23.0 software (SPSS Incorporated, Chicago, IL, USA) Differences were considered significant at the *P* < 0.05.

## Additional files

Additional file 1: **Figure S1.** Morphology and gene expression of known biomarkers in bovine adipocytes. A. Representative image of optical microscope of adipocytes, scale bar = 20 μm. B. Representative image of oil red O staining of adipocytes, scale bar = 20 μm. C. Gene expression of known biomarkers in bovine adipocytes. (TIF 2147 kb)

Additional file 2: The ARRIVE Guidelines Checklist. (PDF 1067 kb)

## Abbreviations

ADPN: Adiponectin
ATRA: All-trans retinoic acid
C/EBPα: CCAAT/enhancer-binding protein-α
IL-10: Interleukin 10
IL-17: Interleukin 17
IL-1β: Interleukin 1 beta
IL-6: Interleukin 6
LPS: Lipopolysaccharide
NEB: Negative energy balance
PLIN1: Perilipin 1
PPARγ: Peroxisome proliferator-activated receptor gamma
RA: Retinoic acid
SV cells: Stromal vascular (SV) cells
TGFβ1: Transforming growth factor beta 1
TGFΒR1: TGF-beta type I receptor
TGFΒR2: TGF-beta type II receptor
TNF-α: Tumor necrosis factor alpha
